# Evidence for a universal saturation profile for radial viscous fingers

**DOI:** 10.1038/s41598-019-43728-z

**Published:** 2019-05-23

**Authors:** Tim H. Beeson-Jones, Andrew W. Woods

**Affiliations:** BP Institute, University of Cambridge, Cambridge, CB3 OEZ England

**Keywords:** Hydrology, Fluid dynamics

## Abstract

Complex fingering patterns develop when a low viscosity fluid is injected from a point source into the narrow space between two parallel plates initially saturated with a more viscous, immiscible fluid. We combine historical and new experiments with (a) a constant injection rate; (b) a constant source pressure; and (c) a linearly increasing injection rate, together with numerical simulations based on a model of diffusion limited aggregation (DLA), to show that for viscosity ratios in the range 300–10,000, (i) the finger pattern has a fractal dimension of approximately 1.7 and (ii) the azimuthally-averaged fraction of the area occupied by the fingers, *S*(*r*,*t*), is organised into three regions: an inner region of fixed radius, *r* < *r*_b_, which is fully saturated with injection fluid, *S* = 1; a *frozen finger* region, *r*_b_ < *r* < *r*_f_ (*t*), in which the saturation is independent of time, *S*(*r*) = (*r*/*r*_b_)^−0.3^; and an outer *growing finger region*, *r*_f_(*t*) < *r* < 1.44 *r*_f_(*t*), in which the saturation decreases linearly to zero from the value *(r*_f_*/r*_b_)^−0.3^ at *r*_f_*(t)*. For a given injected volume per unit thickness of the cell, *V* ≫ π*r*_b_^2^, we find *r*_f_ = 0.4*r*_b_ (*V*/*r*_b_^2^)^1/1.7^. This apparent universality of the saturation profile of non-linear fingers in terms of the inner region radius, *r*_b_, and the injected volume *V*, demonstrates extraordinary order in such a complex and fractal instability. Furthermore, control strategies designed to suppress viscous fingering through variations in the injection rate, based on linear stability theory, are less effective once the instability becomes fully nonlinear.

## Introduction

If air is injected from a point source into a second immiscible fluid confined within a narrow planar channel, then, with a constant injection rate, the interface eventually becomes unstable and a series of viscous fingers develop^1^. Linear stability theory suggests that the fingering process may be suppressed if the injection rate is varied with time, with the optimal profile involving a linearly increasing injection rate^2^. However, it is not known whether there is any benefit to such an approach once the finger pattern has developed into a fully nonlinear regime. The lack of a simple model for the non-linear fingering process makes it challenging to address such questions. Classical experiments^3^ have shown that the nonlinear finger pattern has a fractal dimension of 1.70 ± 0.01, and simulations with diffusion limited aggregation (DLA) models^4^ have replicated many of the features of viscous fingering in cases with relatively large flow rates. Guided by this historical work, we now explore the impact of changes in the injection rate with time on the structure of the finger pattern and, in particular, the variation of the saturation with radius.

## Results

**Figure 1:**
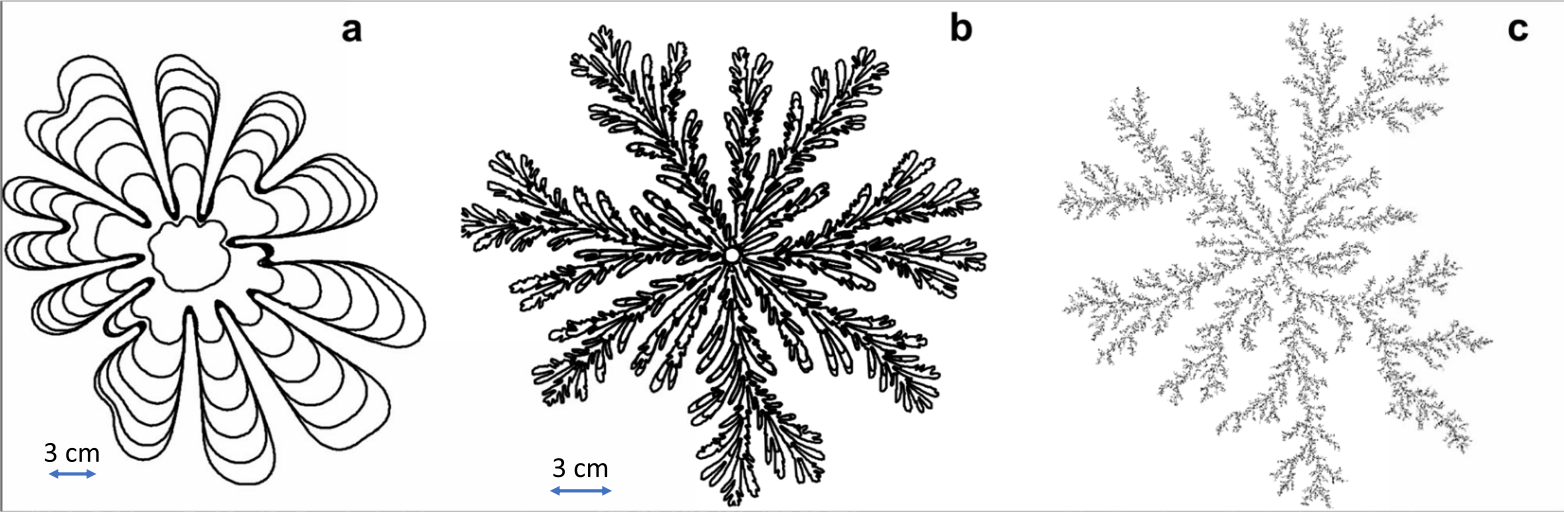
Illustration of fingering patterns. (**a**) Viscous fingering experiment C1 (see Table in supplementary information), with injection of air at a constant flow rate and intervals Δt = 1 s. (**b**) Viscous fingering experiment P3, with air injection at a constant pressure: ΔP = 1.25 atm, Δt = 0.5 s, from ref.^3^. (**c**) DLA numerical model calculation showing the cluster which forms when 10^5^ particles move inwards, in sequence, on random walks; each particle which collides with the cluster sticks and causes it to grow.

We have analysed the output from (a) a series of new DLA calculations, (b) many of the classical radial viscous fingering experiments^3,5–8^ and (c) a series of new experiments, some of which have injection rate which varies with time: each experiment analysed is listed in the Table in the supplementary information. In Fig. 1a, we illustrate a typical nonlinear viscous fingering pattern: this was obtained from an experiment we performed with constant-flow injection. In Fig. 1b we show the pattern from an experiment with constant-pressure injection^3^, which involves much more tip-splitting than in Fig. 1a. Measurement of the area of the fingers as a function of time from data supplied by the authors^3^ shows that in this case the injection rate evolves approximately as $Q={Q}_{0}{t}^{0.6}$. In Fig. 1c we illustrate the pattern produced by a DLA calculation using an off-lattice algorithm^9^, which replicates many features of the nonlinear fingering process.

**Figure 2:**
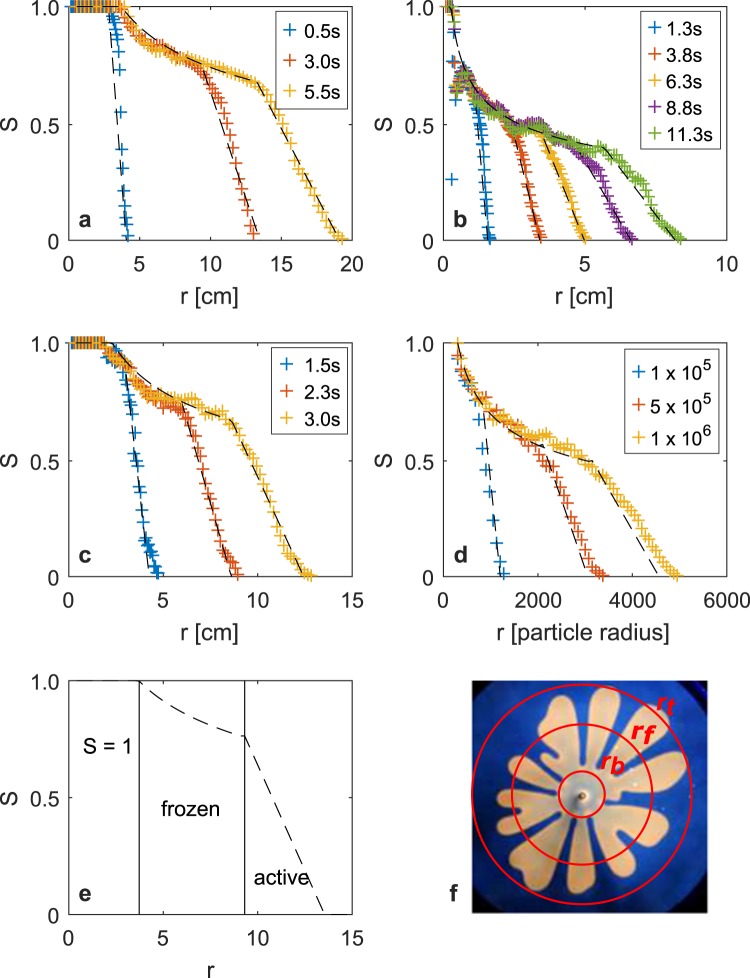
(**a**–**d**) The measured saturation profiles (crosses) as a function the radius. In each case, the saturation model (developed below) is shown by the black broken lines. (**a**) Time series of saturation profiles for the case of constant flow with forcing C* = 12 (see eqn 5 later in text) and gap *b* = 1.5 mm; the profiles are separated by an interval of 2.5 s. (**b**) Time series of saturation profiles with constant pressure injection, Δ*P* = 1.25 atm (ref.^3^), with profiles separated by an interval of 2 s. (**c**) Time series of saturation profiles for injection with a linearly increasing flow rate with forcing C* = 0.19 (eqn 5) and gap width *b* = 1.0 mm, with profiles separated by an interval of 0.75 s. (**d**) Saturation profiles of a particle cluster formed in a numerical DLA calculation with the number of particles released for each profile shown in the legend. Here the radius is scaled with the particle radius. (**e**) A cartoon to illustrate the three regions in the saturation profile, and the bounding radii *r*_b_ < *r*_f_ < *r*_t_. (**f**) Circles with the bounding radii *r*_b_, *r*_f_, and *r*_t_ superimposed over a photograph of the fingers in an experiment.

In order to characterize the fingering pattern, for each experiment we have computed the area covered by fingers, 2π *r S*(*r,t*) d*r*, in the annular ring bounded by the circles with radii *r* and *r* + d*r*, with d*r* ≪ *r*. This reveals the variation of saturation *S*(*r*,*t*) with radius at a series of times *t*. Figure 2 shows the saturation profile for three experiments, corresponding to: (a) constant flow injection, (b) constant pressure injection and (c) approximately linearly increasing injection as well as (d) DLA calculations. In each case, the data are accompanied by a model saturation profile (black broken lines), which we now develop and explain. We note that since the oil preferentially wets the glass in favor of the air, there is a thin film of liquid which remains on the walls of the cell as the fingers advance, but this film is much thinner than the gap width^10,11^. The thickness of the film scales with *b*(*μU*/*σ*)^2/3^^11,12^, where *b* is the cell thickness, *μ* and* U* the fluid viscosity and speed, and *σ* the surface tension, and this has value of order 0.01*b*, suggesting that the residual liquid film is very thin relative to the gap width. This is consistent with estimates of the injected volume of air based on the product of the gap width and the finger area, which agree within measurement error with the actual volume injected.

As may be seen in Fig. 2 (panels a–f), we find that three distinct regions develop: (i) a central *fully flooded* region, *r* < *r*_b_, in which* S* = 1, where *r*_b_ depends on the injection rate but is approximately independent of time, (ii) an inner *frozen finger* zone, *r*_b_ < *r* < *r*_f_(*t*), where the saturation is independent of time and gradually decays with radius, and (iii) an *active outer finger* zone, *r*_f_ (*t*) < *r* < *r*_t_(*t*), in which the fingers advance in time but also thicken and undergo tip-splitting events. Historical data have shown that the viscous fingering pattern is fractal with dimension *D* = 1.7. If the saturation in the frozen finger region decays with radius as

1a $$S(r)=\,{(\frac{r}{{r}_{b}})}^{D-2},$$

then for *r*_f_  $\gg$  *r*_b_, integration shows that this saturation profile is consistent with the region covered by the frozen finger pattern being fractal (see eqn 2 below).

Observations of the saturation in the finger patterns shown in Fig. 2 suggest that in the evolving *outer finger* zone, *r*_f_ (*t*) < *r* < *r*_t_(*t*), the saturation decreases to zero approximately linearly with radius. This suggests a simple model for the saturation in this region given by

1b $$S(r,t)=S({r}_{f},t)(\frac{{\rm{\Lambda }}\,-\,r/{r}_{f}}{{\rm{\Lambda }}\,-\,1}),$$

here, the outer radius of the finger zone, *r*_t_(*t*), corresponds to the minimum radius at which *S*(*r*, *t*) = 0, and we define ${\rm{\Lambda }}$ according to the relation *r*_t_ = ${\rm{\Lambda }}$*r*_f_.

Integration of the model saturation profile across the frozen finger zone and the advancing front region leads to the approximate relation

2 $${(\frac{{r}_{f}}{{r}_{b}})}^{D}=\,{(2\pi (\frac{1}{D}+\frac{({\rm{\Lambda }}-1)({{\rm{\Lambda }}}^{3}-{\rm{\Lambda }}-1)}{{\rm{\Lambda }}}))}^{-1}\frac{V(t)}{{{r}_{b}}^{2}},$$

which is valid provided that *V(t)*, the invaded area per unit thickness of the cell at time *t*, satisfies *V*(*t*) ≫ *πr*_b_^2^. In order that this expression represents a fractal shape for all values of *r*_f_(*t*), with a constant value of *D*, we expect that $\Lambda$ is also a constant.

By comparing the saturation profile for the DLA calculations with eqn (1) at a series of times, we find that the best fit values for the model with fractal dimension *D* = 1.7^3,4,9^ are ${\rm{\Lambda }}$ = 1.43 ± 0.04 and *r*_b_ = 0.14 ± 0.01, where lengths have been scaled with the radius of the particle. To find the best fit, we minimise the quantity:

3 $${E}=\frac{\int |{S}_{model}-S|2\pi rdr}{\int S\,2\pi rdr}.\,$$

**Figure 3:**
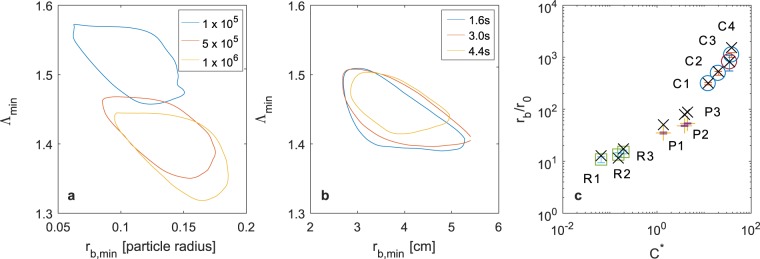
Fitting the model parameters. (**a**,**b**) Locus of the points in (*r*_b_, Λ) space which give rise to a 10% error in the prediction of the saturation profile (eqn 3), *E* = 0.1. For points within each curve, the error is less than 10% and so this provides an estimate of the error bars in fitting the model to the data. (**a**) Diffusion limited aggregation calculations with the number of particles released shown in the legend. The best-fit parameters converge after 5 × 10^6^ particles have been released. (**b**) A constant flow rate experiment leading to viscous fingering (C2 in key below) with the error calculated at three times after the start of injection as shown in the legend. (**c**) The value of *r*_b_ for various experiments as calculated from images of the finger pattern at which the saturation first falls below unity (black crosses) and as calculated from the best fit of the saturation profile to the model (eqn 1). The two estimates as very similar. The error bars on the fitted values correspond to the range of *r*_b_ in which *E* < 0.1. Experiment Key. Constant flow: **C1:** forcing C* = 12; gap spacing *b* = 1.5 mm, **C2:** C* = 20; *b* = 1.5 mm, **C3:** C* = 35; *b* = 1.0 mm, **C4:** C* = 35; *b* = 0.254 mm^†^. Constant pressure^3^: **P1:** pressure difference Δ*P* = 0.25 atm, **P2:** Δ*P* = 0.5 atm, **P3**: Δ*P* = 1.25 atm. Linearly increasing flow: **R1:** C* = 0.065; gap spacing *b *= 1.5 mm, **R2:** C* = 0.15; *b* = 1.0 mm, **R3**: C* = 0.19; *b *= 1.0 mm.

To illustrate the sensitivity of the model to the values of ${\rm{\Lambda }}$ and *r*_b_ we show the contour on which *E* = 0.1, as a function of ${\rm{\Lambda }}$ and *r*_b_ with *D* = 1.7 (Fig. 3a). We have carried out a similar analysis for each of the viscous fingering experiments to determine the best fit values of ${\rm{\Lambda }}$ and *r*_b,_ and in Fig. 3b illustrate the contour *E* = 0.1 for a typical experiment. Repeating this analysis across all our experiments, we find that ${\rm{\Lambda }}$ = 1.44 ± 0.01 for *D* = 1.7.

We have also estimated the value of *r*_b_ for each experiment as determined from (i) the point at which the saturation first decreases below unity (crosses, Fig. 3c), and (ii) the best fit of the data to the model saturation profile (circles; Fig. 3c). The dependence of *r*_b_ on the source parameters is more subtle, but for each experiment a convenient length-scale with which to scale *r*_b_ is the radius *r*_0_ at which the interface first becomes unstable according to linear stability analysis^1^, as given by

4 $${r}_{0}={(\frac{{Q}_{0}}{\pi (1+\alpha )})}^{\frac{\alpha }{3\alpha +1}}{(\frac{{b}^{2}\sigma }{\mu {Q}_{0}})}^{\frac{\alpha +1}{3\alpha +1}}.$$

Figure 3c shows the dependence of this dimensionless radius, *r*_b_/*r*_0_, on the non-dimensional injection parameter, C*, as given by

5 $${C}^{\ast }=\frac{{{Q}_{0}}^{\frac{1}{1+\alpha }}\mu }{{b}^{\frac{1-\alpha }{1+\alpha }}\sigma },\,$$

where the areal injection rate *Q*(*t*) = *Q*_*0*_ *t*^*α*^, *μ* is the viscosity, *b* the cell thickness, and *σ* the surface tension. It is seen that *r*_b_/*r*_0_ increases with C* corresponding to there being a larger fully saturated region in a more intense flow. It is interesting to note that r_b_ $\gg$ r_0_, which indicates that the fully saturated zone extends some distance beyond the radius at which the linear instability initially sets in. This may be expected since at the onset of instability, the interface is still migrating outwards, and as the radius increases, the wavenumber of the most unstable mode also changes^1,2^.

**Figure 4:**
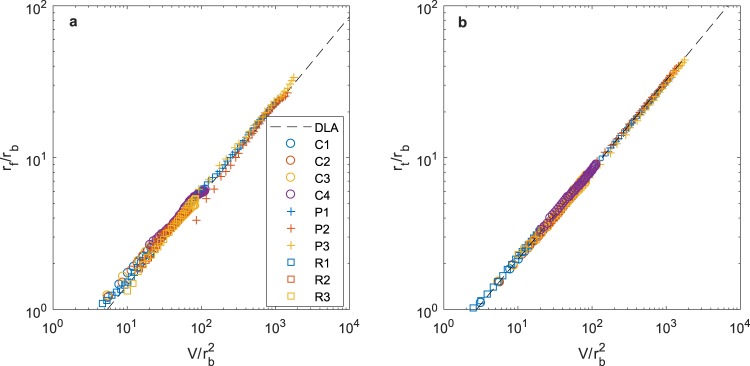
The radii that define the structure of the pattern. Variation of (**a**) the radius of the frozen zone, *r*_f_, and (**b**) the outer radius as a function of the volume injected. The experimental data and the DLA calculation results all collapse to the same radius with time. The different data shown in this figure are labelled in the legend.

As a further test of the model, Fig. 4 illustrates the variation with time of the outer radii of the frozen finger zone, *r*_f_, and the growing finger zone, *r*_*t*_, scaled with the best-fit values of *r*_b_ for each experiment. Data are given for a large number of experiments from both the literature and our new experiments. We have also evaluated *r*_f_/*r*_b_ (eqn 2) using the parameters obtained from the DLA calculations *D* = 1.7, $\Lambda$ = 1.43 and *r*_b_ = 0.14 (black broken lines), where *V* is defined in terms of the number of particles added as *V* = *n*π.

Figure 4 illustrates that, for all experiments analysed, eqn (2) provides a good approximation for the variation of the radii *r*_f_ and *r*_t_ as a function of *V*/*r*_b_^2^, irrespective of whether the experimental injection rate varies in time or is constant, and also for the DLA simulations. This is consistent with Fig. 2 where the model (broken black lines) appears to give good agreement with the evolving saturation profiles for the various injection conditions.

## Discussion

The universality of the model for the variation of the saturation profile with radius, and the recognition that there is a transition from the frozen non-linear finger region to the growing non-linear finger region in the leading part of the flow provide new insight about and a succinct description of the evolution of such nonlinear viscous fingers. In contrast to predictions of linear stability theory^1,2,13^, in which varying the injection rate with time but injecting a fixed volume leads to differences in the amplitude of the most unstable mode^2^, the apparent universality of the non-linear finger pattern, as characterised by the saturation profile shown herein, does not seem to depend on the injection rate history for a given total volume injected.

## Methods

In our experiments, two World Precision Instruments AL1000-220 pumps were used to inject air into a Hele-Shaw cell of radius 30 cm, with plate thickness 1 cm, and gap width of either 1 mm or 1.5 mm set by positioning micrometers. The cell was initially filled with rapeseed oil (*μ* = 50 mPa s; *σ* = 31 mN/m^2^). Another experiment, marked C4 in Fig. 4, was provided by I. Bischofberger *et al*. and used the same setup^10^ but is previously unpublished. It involved the displacement of silicone oil (*μ* = 298 mPa s; *σ* = 27 mN/m^2^) by water. In the DLA simulations, we used code supplied by the co-authors of publication^9^ and used the mean of 7 saturation profiles for each aggregate size.

## Supplementary information

Supplementary information
