# Supplementary material for: Evidence for a universal saturation profile for radial viscous fingers
Source: Sci Rep. 2019 May 23;9:7780. doi: 10.1038/s41598-019-43728-z (PMC6533247; doi:10.1038/s41598-019-43728-z)
Supplement: Supplementary file 1 — Supplementary information [file 41598_2019_43728_MOESM1_ESM.docx]

**Supplementary Material Title Page**

**Evidence for a universal saturation profile for radial viscous fingers**

Tim H. Beeson Jones^1^ and Andrew W. Woods^1^

**Supplementary Information**

Table of experiments performed for this study (invading fluid is air)

|  | *Q_0_*  [cm^2^/s^α+1^] | *α ^(4)^* | *b*  [mm] | *µ*  [Pa s] | *σ*  [N/m] | V^(5)^ | $Ʌ$ ^(^*^1)^* | *r*_b_*^(1)^*  [cm] | *E^(2)^* |
| --- | --- | --- | --- | --- | --- | --- | --- | --- | --- |
| C1 | 110 | 0.0 | 1.5 | 0.050 | 0.031 | 2500 | 1.453±0.003 | 3.82±0.07 | 3% |
| C2 | 180 | 0.0 | 1.5 | 0.050 | 0.031 | 2500 | 1.450±0.003 | 3.70±0.07 | 5% |
| C3 | 230 | 0.0 | 1.0 | 0.050 | 0.031 | 2500 | 1.452±0.004 | 2.92±0.06 | 5% |
| R1 | 21 | 1.1 | 1.5 | 0.050 | 0.031 | 2500 | 1.446±0.006 | 3.8±0.2 | 8% |
| R2 | 52 | 0.8 | 1.0 | 0.050 | 0.031 | 2500 | 1.446±0.002 | 2.38±0.05 | 5% |
| R3 | 52 | 0.9 | 1.0 | 0.050 | 0.031 | 2500 | 1.453±0.004 | 2.2±0.2 | 5% |
| DLA |  |  |  |  |  |  | 1.43±0.04 | 0.14±0.01^(3)^ | 11% |

Table of Experiments from the literature (various invading fluids)

|  | *Q_0_*  [cm^2^/s^α+1^] | *α ^(4)^* | *b*  [mm] | *µ*  [Pa s] | *σ*  [N/m] | V^(5)^ | $Ʌ$ ^(^*^1)^* | *r*_b_*^(1)^*  [cm] | *E^(2)^* |
| --- | --- | --- | --- | --- | --- | --- | --- | --- | --- |
| C4† | 8.0 | 0.0 | 0.254 | 0.297 | 0.027 | 300 | 1.441±0.001 | 0.61±0.01 | 6% |
| P1 (Ref[3]) | 2.7 | 0.5 | 0.127 | 0.345 | 0.021 | 2x10^4^ | 1.43±0.01 | 0.27±0.01 | 5% |
| P2  (Ref[3]) | 7.4 | 0.4 | 0.127 | 0.345 | 0.021 | 2x10^4^ | 1.44±0.01 | 0.20±0.01 | 6% |
| P3  (Ref[3]) | 27 | 0.6 | 0.127 | 0.345 | 0.021 | 2x10^4^ | 1.43±0.01 | 0.21±0.02 | 9% |
| Ref [8]  Fig 17a |  |  |  |  |  |  | 1.434 |  | 11% |
| Ref [8]  Fig 17b |  |  |  |  |  |  | 1.445 |  | 6% |
| Ref [6]  Fig 12 |  |  |  |  |  |  | 1.446 |  | 7% |
| Ref [5]  Fig 1 |  |  |  |  |  |  | 1.458 |  | 5% |
| Ref [7]  Fig 1 |  |  |  |  |  |  | 1.46 |  | 8% |

1. Where movies were available, the value of $Ʌ$ was evaluated for a large number of frames and the value presented is the mean and the error is the standard deviation;
2. Evaluated with parameter value $Ʌ$ = 1.44
3. Unit of length for the DLA calculations is the particle radius
4. For experiments P1-P3 and R1-R3, the value of α is calculated by fitting a power law to measurements of the fluid area as a function of time, based on movies of the experiment.
5. Viscosity ratio between the defending and invading fluids.

† Provided by I. Bischofberger and the authors of [10] (water is the invading fluid).
